# Assessment of Duration of Staying Free from Acquiring Rehappening Opportunistic Infections among Pre-ART People Living with HIV/AIDS between 2008 and 2013

**DOI:** 10.1155/2015/146306

**Published:** 2015-01-18

**Authors:** Habtamu Mellie Bizuayehu, Direslgne Misker Abyu, Amlaku Mulat Aweke

**Affiliations:** ^1^Department of Public Health, College of Medicine and Health Science, Debre Markos University, 269 Debre Markos, Ethiopia; ^2^Department of Public Health, College of Health Science, Arba Minch University, Arba Minch, Ethiopia; ^3^Department of Midwifery, College of Health Science, Mekelle University, Mekelle, Ethiopia

## Abstract

*Introduction.* In regional state of the study area, HIV (Human Immunodeficiency Virus) prevalence is 2.2% and opportunistic infections (OIs) occurred in 88.9% of pre-ART (Antiretroviral Therapy) people living with HIV/AIDS (PLWHA). Even though OIs are prevalent in the study area, duration of staying free from acquiring rehappening opportunistic infections and its determinant factors are not studied. *Method*. The study was conducted in randomly selected 341 adult Pre-ART PLWHA who are included in chronic HIV care. OI free duration was estimated using the actuarial life table and Kaplan Meier survival. Cox proportional-hazard model was used to calculate hazard rate. *Result*. OIs were rediagnosed in three quarters (75.37%) participants. In each week the probability of getting new recurrence OI was about 15.04 per 1000 person weeks. The median duration of not acquiring OI recurrence was 54 weeks. After adjustment, variables associated with recurrence were employment status, marital status, exposure for prophylaxis and adherence to it, CD4 count, and hemoglobin value. *Conclusion*. Giving prophylaxis and counseling to adhere it, rise in CD4 and hemoglobin level, and enhancing job opportunities should be given for PLWHA who are on chronic HIV care while continuing the care.

## 1. Introduction

Globally approximately 34 million people were living with HIV in 2011 [1, 2]. Still, there were about 2.2 million new infections [3]. Since the beginning of the epidemic nearly 30 million people have died of AIDS (Acquired Immunodeficiency Syndrome) related causes [1, 2, 4].

About 22.9 million which is 67% of those living with HIV/AIDS globally are in Africa though only about 12% of the world's population lives in the region [2]. In terms of mortality, the region represents about 79% of AIDS mortality globally [5].

According to 2011 Ethiopian demographic health survey the overall national adult HIV prevalence is 1.5%. The survey showed the HIV prevalence was 2.2% in Amhara region which is found in North West Ethiopia [6].

Human Immunodeficiency Virus (HIV) infection leads to acquired immunodeficiency syndrome (AIDS) and major causes of morbidity and mortality of such patients are OIs [7]. OIs can occur in up to 40% of PLWHA with a CD4 count less than 250 cells/mm^3^ [8].

A national study in Ethiopia showed HIV patients' had OIs like Herpes Zoster scar (19.3%), pulmonary tuberculosis (5.2%), and pneumonia (5.2%) [9]. The respective prevalence of OIs in pre-ART HIV patients' in two studies in Northwest Ethiopia was 88.9% [10] and 82.4% [11].

The problem of HV and OIs is still high in the study area though there is no prior local evidence in the study area thus the current study would give the duration of staying free from acquiring rehappening opportunistic infections after its treatment and its determinant factors. The output of the study will be used to plan resources needed for chronic HIV/AIDS care and to know groups of PLWHA given especial attention during care. The evidence is expected to be used by governmental and nongovernmental organizations working on HIV/AIDS or mainstreaming it in order to inform policy makers and medical practitioners.

## 2. Methods and Materials

### 2.1. Study Setting and Source Population

The study was conducted in Debre Markos town public health institutions among adult Pre-ART PLWHA included to chronic HIV care between 25 March 2008 and 24 March 2013. Debre Markos town is found 299 kilometer away from Addis Ababa (a capital city of Ethiopia) and it has one referral hospital, three public health centers, two NGO clinics, and ten other private clinics and though only the referral hospital and one health center providing chronic HIV care for the HIV/AIDS patients. Thus we conduct a study using retrospective cohort study design on health institutions providing chronic HIV care in the town. The source populations were all adult with age above 17 years PLWHA who had chronic HIV care in the town public health institutions. PLWHA who were having incompletely documented follow up format; not developing OI while registered on HIV chronic care; not taking standard treatment after developing OI according to the Ethiopian Ministry of Health guideline; and pre-ART pregnant or lactating mothers who were taking zidovidine for prevention of mother to child transmission of HIV/AIDS were excluded from the study.

### 2.2. Sampling and Data Collection Procedure

The sample size was calculated based on the two-sided 95% confidence interval and 3.5% margin of error and by using the proportion of pre-ART HIV patients' having OI in Northwest Ethiopia study, which was 88.9% [10]. The calculated sample size using Open-Epi Version 2.3 May 2009 was 310 then after adding 10% contingency the final was 341.

About 2712 PLWHA who fulfill the inclusion criteria were requited from the already available list of PLWHA who were on chronic HIV care in ART clinics. And then selection of participants was made by applying simple random sampling procedure using random number table. The needed data is available on study participants' treatment card and chronic HIV care follow-up form which it is found in the ART clinic but rarely they may seek treatment out-off their follow-up clinic. Thus in order to reduce falsely survival increment, study participants were asked by data collectors about treatment history out of the follow-up health institution and for those doing it, the treatment was checked and abstracted in the respective health institutions. Seeking treatment out of the follow-up health institution was asked when PLWHA come to health institution for follow-up or treatment or by using registered address on follow-up form like phone number or kebele, house number which was used to get to them.

Data collection instrument was developed from federal ministry of health chronic HIV care follow-up form which is used in the ART clinic and also the patient's card. The data was collected by reviewing chronic HIV care follow-up form and patients' card. Among a serious of laboratory measurements (like CD4 count, hemoglobin value, height, and weight) the most nearest to the study period were taken as baseline characteristics. And among the serious measurements performed on PLWHA while she/he is included on the study, the nearest to the OI recurrence or censored was taken as the end line or follow-up values.

Approval of OI free duration was done by reviewing chronic HIV care follow-up form or patient card in ART clinic or out of ART clinic if study participants seek treatment out of ART clinic.

Study participants who start ART/drop-out/loss follow-up/transferred out/dead by any disease other than OI/cause of death not confirmed while on study or not developing OI at end of the study period were censored. A selected and trained health professionals working in ART clinics in each health institution were used as data collectors and supervisors.

### 2.3. Operational Definition

*Survival*. Duration of free of OI rehappening.

*Censored*. Nonrelapse of OI in study participant during follow-up on study, but future relapse is uncertain.

Recurrence/rehappen/relapse: happening or diagnosis of any type of OIs by health personals working in ART clinic after completing the preceding treatment of any type of OI.

*Drop Out*. If a PLWHA on HIV care lost to follow-up for more than three months as recorded by ART health personnel.

*Lost to Follow-Up*. If PLWHA on HIV care not seen for equal to or more than one month as recorded by ART health personnel.

*Transferred out.* If PLWHA on HIV care in one health institution shift to other health institution.

*Good Adherence*. If PLWHA adherent ≥95% that is the percentage of missed dose is <2 doses of 30 doses or <3 dose of 60 dose as documented by ART health personnel.

*Fair Adherence*. If PLHIV adherent 85–94% that is the percentage of missed dose is 3–5 doses of 30 doses or 3–9 dose of 60 dose as documented by ART health personnel.

*Poor Adherence*. If PLHIV adherent <85% that is the percentage of missed dose is ≥6 doses of 30 doses or >9 dose of 60 dose as documented by ART health personnel.

### 2.4. Data Quality Management and Statistical Analysis

To maintain data quality training was given for data collectors and for supervisors. Properly designed data collection material was developed from Ethiopian federal ministry of health chronic HIV care follow-up form and patients' card. To check correct data collection 10% of the sample was reabstracted by supervisors. The data were double entered by trained data clerk to check correct data entry. After completing data entry, outliers and any missed values were checked using frequency, listing, and sorting and any identified error at any step was corrected by revising the original data abstraction format.

After coding each abstraction format, data was entered in to Epi Info version 3.5.1 statistical package. Analysis of data was done using Open-Epi Version 2.3 May 2009, SPSS version 20, and STATA version 11 statistical packages.

Incidence rate was calculated by dividing total events to person-weeks. OI free duration was estimated using the actuarial life table and Kaplan Meier survival. Assumption of proportional-hazard was checked by Schoenfeld residual with *P* value ≥0.1 (*α* = 10%) and the assumption was not violated. Multicollinearity was checked using Pearson correlation, tolerance/variance inflation factor and there was no colinearity To determine independent predictors of OI free duration cox proportional-hazard model was used to calculate the hazard rate. Variables having *P* value <0.05 at bivariate analysis and not collinear were entered in multivariate cox proportional hazard model to determine the adjusted hazard rate. The cut-off point for significant association was *P* value 0.05.

### 2.5. Ethical Consideration

Ethical approval and clearance was given by School of Public Health Addis Ababa University ethical committee. Permission was also obtained from the concerned bodies of East Gojam zone and Debre Markos town Health Department and the responsible bodies of hospital and health centers. To maintain confidentiality of PLWHA, health professionals working in ART clinic were abstracting the data. In addition no personal identifier was extracted on medical records and the recorded data was not accessed by a third person.

## 3. Result

In the five year study period among 341 study participants, the median duration of follow-up was 41 weeks (95% CI: 37–47.97) and the minimum, maximum, and interquartile range of follow-up was 1, 234, 50 weeks, respectively. Among the study participants majority of them were females 234 (68.6%), orthodox Christian 316 (92.7%), living in urban 229 (67.2%), not educated 153 (44.9%), married 130 (38.1%), and not employed in governmental or private organizations 291 (85.3%). Their mean age was 33.3 (±10.6) years, in which almost all of them were below 50 years old 318 (93.3%) (Table 3).

### 3.1. The Baseline and Follow-Up Laboratory, Clinical and Prophylaxis Characteristics

At base line, the median values for CD4 count (cells/uL) and hemoglobin value (g/dL) were 383 and 11.6, respectively and the respective end line values were 382.5 and 12.5. The base line and end line body mass index mean values were 19.1 (±3.1) and 19.7 (±3.1) kilogram per meter square units, respectively.

At start of the study, majority of participants were having WHO stage II OI 165 (48.4%). About 11 (3.2%) participants were having concomitant chronic diseases like hypertension, cardiac disease, and diabetes mellitus. With regard to functional status, almost all of them were working both at base line 83 (83%) and at end line 297 (87.1%) (Table 3).

About three quarters of participants were taking prophylaxis both at base line 244 (71.6%) and at follow-up 255 (74.8%) and almost all of them were having good drug adherence both at base line 225 (92.2%) and at follow-up 231 (90.6%) in which nearly all of them were taking cotrimoxazole both at base line 225 (92.2%) and at follow-up 243 (95.3%) (Table 3).

### 3.2. Incidence of Recurrence and OI Free Duration

The cumulative incidence of OI recurrence was 75.37% (95 CI: 70.6–79.7%) and incidence rate was 15.04 (95 CI: 13.1–16.97%) per 1000 person weeks. Of recurrence OIs, about 12.8% (95 CI: 9.16–17.36%) were self-relapsed and incidence rate of self-relapse was 1.93 (95 CI: 1.35–2.68%) per 1000 person weeks. The most rediagnosed OI was recurrent upper respiratory tract infection 44 (17.1%) whereas chronic diarrhea was most self-relapsed OI (23.7%) (Table 1).

**Figure 1:**
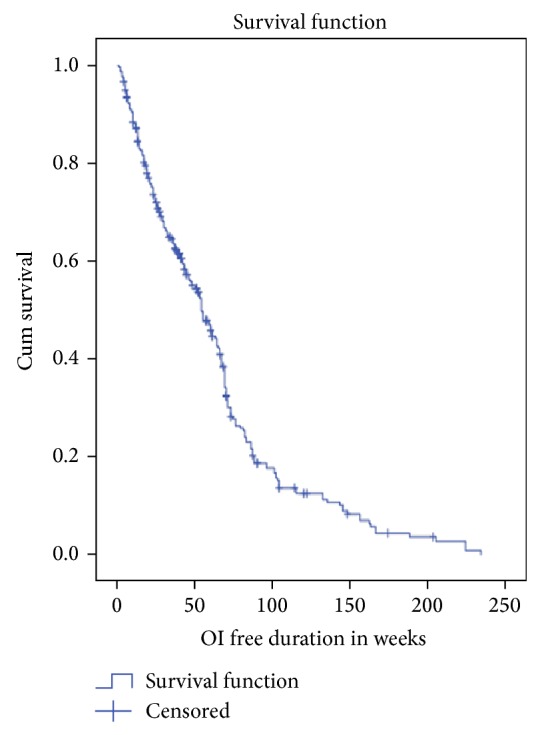
Kaplan-Meier survival estimation of progressing to OI rediagnosing among pre-ART PLWHA in Debre Markos town between 2008 and 2013.

**Figure 2:**
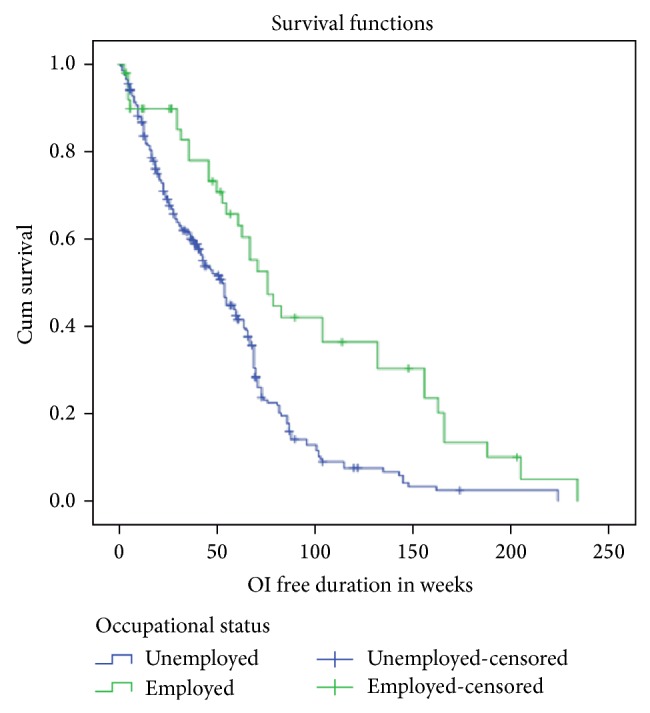
Kaplan-Meier survival estimation of progressing to acquiring OI recurrence among employed and unemployed pre-ART PLWHA in Debre Markos town between 2008 and 2013.

According to the Kaplan-Meier survival estimation, the median duration of not acquiring OI recurrence was 54 weeks (95% CI: 46.9–61.1) (Figure 1). Among participants, those employed were more surviving than unemployed (Figure 2). As the actuarial life table analysis showed about 91% participants were not acquiring OI at end of 10 weeks and the probability of free of OI recurrence at end of 220 and 230 weeks was about 1% and <0.01%, respectively (Table 2).

In bivariate cox proportional hazard model, the predictor variables that showed significant (*P* < 0.05) association with the outcome variable were marital status, occupational status, educational status, the base line and follow-up functional status, having exposure for prophylaxis at baseline and adhering to it both at baseline and at follow up, baseline hemoglobin value, follow-up CD4 count, follow-up body mass index, number of OIs diagnosed at one time at start of the study, and being diagnosed wasting syndrome and Herpes Zoster at start of study (Table 3).

After adjustment for potential confounders in multivariate cox proportional hazard model, the significant (*P* < 0.05) predictors preventing repeated diagnosing of OI rediagnosis were being employed in governmental or private sectors, divorced than married, taking prophylaxis at baseline, having a follow-up CD4 count above 100 cells/*μ*L, and having hemoglobin value of 10 g/dl and above, whereas not adhering to prophylaxis both at base line and at follow-up was the risk factors for short time rediagnosing of OIs (Table 3).

## 4. Discussion

In the five-year study period, the cumulative incidence of OI recurrence was seen in about three quarters (75.37%) of participants. Different studies have diverse figures with regard to the proportion of OI. In North India, tuberculosis was the commonest OI (71%) followed by candidiasis (39.3%),* PCP* (7.4%), cryptococcal meningitis, and cerebral toxoplasmosis (3.7% each) [12]. In the same country of southern India, proportion of pulmonary tuberculosis was (14%) [13]. A national study in Ethiopia showed HIV patients' had OIs like Herpes Zoster scar (19.3%), pulmonary tuberculosis (5.2%), and pneumonia (5.2%) [9]. In Northwest Ethiopia, about 88.9% pre-ART HIV patients had OIs [10]. Another study in similar area also showed that 82.4% pre-ART HIV patients have OIs [11]. The respective prevalence of pulmonary tuberculosis and cryptococcal meningitis among PLWHA in North West Ethiopia was about 7.5% [14] and 8.3% [15]. In about a quarter (22.7%) of PLWHA, chronic diarrhea was seen in Southern Ethiopia [16]. The cumulative incidence of recurrent OIs like recurrent upper respiratory tract infection, chronic diarrhea, bacterial pneumonia, oral candidiasis, herpes zoster, extra pulmonary tuberculosis, PCP, and pulmonary tuberculosis in current study was 17.1%, 14.8%, 9.8%, 9.8%, 9.3%, 9.3%, and 7%, 5.4%, respectively, and this finding is relatively in line with some figures of OIs in the studies [9, 10, 12–14] though it is lower than some other studies [11, 15, 16]. The possible reasons for discrepancy would be different in study design (the prior ones that are cross-sectional), study population (prior ones using PLWHA when coming to initiate ART which will increase the prevalence since ART is initiated using WHO stage of disease and CD4 count), study area, and other sociocultural practices.

In this study, chronic diarrhea was most self-relapsed OI (23.7%) and this might be attributed by not using improved drinking water source and sanitation facility since only 50.8% and 8.8% of Ethiopians were using improved drinking water source and sanitation facility according to 2011 Ethiopian demographic health survey [6].

The current finding of being employed in governmental or private sectors as increasing duration of frequent vising of health institutions due to illness of rehappened OIs was in harmony with a cohort study in United States [17].

The current study that comes up as having a follow-up CD4 count above 100 cells/*μ*L compared to ≤100 cells/*μ*L was preventing repeated diagnosing of OI recurrence and this is in agreement with other studies [18–22]. The HIV cohort study in Switzerland showed that CD4 count is one of the predictor for OI progression; a rise in CD4 count by 50 × 10^6^/L or more by 6 months reduced subsequent OIs with hazard ratio of 0.32 [19]. Another cohort study also showed that higher CD4 cell count was associated with a reduction of risk of new OI progression and with a hazard ratio compared to 100 cells/mL of 0.35 for counts 200 cells/mL, 0.81 for counts 200 to 350 cells/mL, 0.74 for counts 350 to 500 cells/mL, and 0.96 for counts 500 cells/mL or above [22].

In this study, taking prophylaxis at baseline was enhancing duration of frequent diagnosis of recurrent OI and the finding was supported by other studies [23–26]. Primary prophylaxis with trimethoprim-sulfamethoxazole is preventing life-threatening OIs like PCP, toxoplasmosis, and bacterial infections [23]. Taking cotrimoxazole reduces the risk of PCP and tuberculosis [24]. Taking cotrimoxazole prophylaxis was preventing OIs like diarrhea in an experimental study in Ugandan PLHIV adults [25]. The evidence of preventing OIs like PCP using Cotrimoxazole was also assured in an experimental study [26].

## 5. Conclusion and Recommendation

During the historical follow-up period, OIs were rediagnosed in about three quarters (75.37%) of participants. Of rediagnosed OIS, nearly one every ten rediagnosis was self-relapsed (12.8%). In each week the probability of getting new recurrence of any type OI and self-relapse OI was about 15.04 and 1.93 per 1000 person weeks, respectively. Commonly rehappening OIs were recurrent upper respiratory tract infection (17.1%), chronic diarrhea (14.8%), bacterial pneumonia (9.8%), oral candidiasis (9.8%), Herpes Zoster (9.3%), and extra pulmonary tuberculosis (9.3%).

According to the Kaplan-Meier survival estimation, the median duration of not acquiring OI recurrence was 54 weeks. After adjustment for potential confounders in multivariate cox proportional hazard model, the significant (*P* < 0.05) predictors preventing repeated diagnosing of OI recurrence were being employed in governmental or private sectors, divorced than married, taking prophylaxis at baseline, having a follow-up CD4 count above 100 cells/*μ*L and having hemoglobin value of 10 g/dL and above, whereas not adhering to prophylaxis both at base line and at follow-up was the risk factors for frequent diagnosing of OI recurrence.

### 5.1. Based on This Study Finding, the Following Recommendations Can Be Forwarded

- Providing prophylaxis and counseling to adhere to it should be further enhanced.
- Treatment and other sportive measures should be given to enhance the CD4 count and hemoglobin value.
- During giving of HIV chronic care especial attention should be given for those not adhering to prophylaxis drug since they have OI recurrence in short periods.
- Governmental or nongovernmental organizations should give especial criteria that support PLWHA to win in computation of job at vacancies since being employed reduces duration repeated rediagnosing of OI.
- Finally we recommend further observational studies with prospective design to ascertain the current findings.

## Figures and Tables

**Table 1:** The rediagnosed OIs of any type and self-relapse among participants' in Debre Markos town between 2008 and 2013.

Rediagnosed OI	Frequency of overall rediagnosis (%)	Self-relapse	
		Yes (%)	No (%)
Recurrent upper respiratory tract infection	44 (17.1)	9 (20.5)	35 (79.5)
Chronic diarrhea	38 (14.8)	9 (23.7)	29 (76.3)
Oral candidacies	25 (9.7)	1 (4)	24 (96)
Pneumonia	25 (9.7)	4 (16)	21 (84)
Herpes Zoster	24 (9.3)	3 (12.5)	21 (87.5)
Extra pulmonary tuberculosis	24 (9.3)	1 (4.2)	23 (95.8)
Minor mucocutanous manifestation	15 (5.8)	2 (13.3)	13 (86.7)
Wasting syndrome	9 (3.5)	2 (22.2)	7 (77.8)
Persistent generalized lymphadenopathy	8 (3.1)	1 (12.5)	7 (87.5)
Viral infection	6 (2.3)	1 (16.7)	5 (83.3)
Others	39 (15.2)	0	39 (100)

Total	257 (100)	33 (12.8)	224 (87.2)

**Table 2:** The actuarial life table estimation of participants' duration of diagnosing of rehappening OI in Debre Markos town between 2008 and 2013.

Interval start time	Number entering interval	Number withdrawing during interval	Number exposed to risk	Number of OIs diagnosed at interval	Cumulative proportion surviving at end of interval	Hazard rate
0	341	7	337.5	32	0.91	0.01
10	302	11	296.5	41	0.78	0.01
20	250	9	245.5	31	0.68	0.01
30	210	11	204.5	20	0.61	0.01
40	179	11	173.5	18	0.55	0.01
50	150	10	145.0	21	0.47	0.02
60	119	8	115.0	31	0.34	0.03
70	80	7	76.5	18	0.26	0.03
80	55	1	54.5	15	0.19	0.03
90	39	2	38.0	2	0.18	0.01
100	35	1	34.5	8	0.14	0.03
110	26	1	25.5	2	0.13	0.01
120	23	2	22.0	0	0.13	0.00
130	21	0	21.0	3	0.11	0.02
140	18	1	17.5	4	0.08	0.03
150	13	0	13.0	2	0.07	0.02
160	11	0	11.0	4	0.05	0.04
170	7	1	6.5	0	0.05	0.00
180	6	0	6.0	1	0.04	0.02
190	5	0	5.0	0	0.04	0.00
200	5	1	4.5	1	0.03	0.02
210	3	0	3.0	0	0.03	0.00
220	3	0	3.0	2	0.01	0.10
230	1	0	1.0	1	0.00	0.20

**Table 3:** The cumulative incidence of diagnosing OI, Kaplan Meier estimation of median duration of not acquiring OI recurrence and cox proportional hazard model of the association between characteristics and OI recurrence among PLWHA in Debre Markos town between 2007 and 2013.

Variables	Diagnosis of recurrence OI		Median KMS	CHR (95% CI)	AHR (95% CI)
	Yes (%)	No (%)			
Marital status					
Married	101 (39.3)	29 (34.5)	60	1	1
Single	68 (26.5)	11 (13.1)	46	1.65 (1.21–2.26)	1.19 (0.72–1.96)
Divorced	60 (23.3)	32 (38.1)	64	1.02 (0.74–1.41)	***0.57 (0.33–0.99)***
Widowed	28 (10.9)	12 (14.3)	42	1.69 (1.10–2.59)	1.64 (0.79–3.40)
Educational status					
Not educated	110 (42.8)	43 (51.2)	55	1	1
Grades 1–8	82 (31.9)	16 (19)	41	1.06 (0.79–1.41)	1.17 (0.70–1.94)
Grades 9–12	44 (17.1)	14 (16.7)	55	0.84 (0.59–1.19)	1.38 (0.71–2.68)
Above grade 12	21 (8.2)	11 (13.1)	67	0.49 (0.30–0.80)	1.55 (0.69–3.47)
Occupational status					
Unemployed	221 (86)	70 (83.3)	53	1	1
Employed	36 (14)	14 (16.7)	76	0.48 (0.33–0.69)	***0.34 (0.16–0.71)***
Functional status^B.^					
Working	206 (80.2)	77 (91.7)	60	1	1
Ambulatory/bed-ridden	51 (19.8)	7 (8.3)	32	1.7 (1.25–2.32)	1.59 (0.81–3.12)
Functional status^F.^					
Working	215 (83.7)	82 (97.6)	60	1	1
Ambulatory/bed-ridden	42 (16.3)	2 (2.4)	37	1.65 (1.17–2.31)	0.97 (0.52–1.81)
Number of OIs treated at base line					
1	203 (79)	62 (73.8)	59	1	1
≥2	54 (21)	22 (26.2)	44	1.38 (1.01–1.87)	1.19 (0.69–2.07)
Prophylaxis adherence^B.^					
Good	170 (91.4)	55 (94.8)	64	1	1
Fair	6 (3.2)	0 (0)	43	2.69 (1.19–6.13)	***14.92 (1.03–215)***
Poor	10 (5.4)	3 (5.2)	23	1.76 (0.92–3.35)	***5.96 (1.21–29.41)***
Prophylaxis adherence^F.^					
Good	173 (88.3)	58 (98.3)	64	1	1
Fair	9 (4.6)	0 (0)	55	1.06 (0.54–2.08)	***5.39 (1.77–16.36)***
Poor	14 (7.1)	1 (1.7)	22	2.2 (1.27–3.82)	***5.79 (1.86–17.98)***
Prophylaxis exposure^B.^					
No	71 (27.6)	26 (31)	37	1	1
Yes	186 (72.4)	58 (69)	63	0.64 (0.49–0.85)	***0.31 (0.19–0.49)***
CD4 count (cells/*μ*L)^F.^					
≤100	4 (2.2)	1 (1.4)	17	1	1
101–199	19 (10.6)	3 (4.2)	60	0.19 (0.06–0.58)	***0.12 (0.029–0.49)***
200–350	50 (27.8)	27 (37.5)	69	0.14 (0.048–0.39)	***0.21 (0.057–0.79)***
351–499	58 (32.2)	24 (33.3)	66	0.17 (0.058–0.47)	***0.16 (0.045–0.59)***
≥500	49 (27.2)	17 (23.6)	70	0.13 (0.046–0.38)	***0.17 (0.46–0.62)***
Hemoglobin value (g/dL)^B.^					
<10	61 (31.1)	5 (16.7)	26	1	1
≥10	135 (68.9)	25 (83.3)	53	0.55 (0.40–0.75)	***0.49 (0.25–0.97)***
Body mass index (kg/m^2^)^F.^					
≤18.4	122 (47.7)	36 (42.9)	46	1	1
18.5–22.9	103 (40.2)	35 (41.7)	54	0.93 (0.71–1.21)	0.89 (0.54–1.46)
≥23	31 (12.1)	13 (15.5)	69	0.54 (0.37–0.79)	0.61 (0.33–1.14)
Herpes Zoster diagnosis^B.^					
No	202 (78.6)	73 (86.9)	55	1	1
Yes	55 (21.4)	11 (13.1)	48	1.38 (1.02–1.86)	1.18 (0.65–2.13)
Wasting syndrome diagnosis^B.^					
No	247 (96.1)	84 (100)	55	1	1
Yes	10 (3.9)	0 (0)	23	2.39 (1.21–4.34)	1.29 (0.36–4.71)

^B^Base line value.

^F^Follow-up value.

KMS: Kaplan Meier survival in weeks.

CHR: crude hazard rate.

AHR: Adjusted hazard rate.
